# A common molecular signature of patients with sickle cell disease revealed by microarray meta-analysis and a genome-wide association study

**DOI:** 10.1371/journal.pone.0199461

**Published:** 2018-07-06

**Authors:** Cherif Ben Hamda, Raphael Sangeda, Liberata Mwita, Ayton Meintjes, Siana Nkya, Sumir Panji, Nicola Mulder, Lamia Guizani-Tabbane, Alia Benkahla, Julie Makani, Kais Ghedira, H3ABioNet Consortium

**Affiliations:** 1 Laboratory of Bioinformatics, Biomathematics and Biostatistics, Institute Pasteur of Tunis, Tunis, Tunisia; 2 University of Tunis El Manar, Tunis, Tunisia; 3 Faculty of Science of Bizerte, Jarzouna, University of Carthage, Tunisia; 4 Muhimbili University of Health and Allied Sciences, Dar es Salaam, Tanzania; 5 University of Cape Town, Cape Town, South Africa; 6 Laboratory of Medical Parasitology, Biotechnology and Biomolecules, Institute Pasteur of Tunis, Tunis, Tunisia; The University of Tokyo, JAPAN

## Abstract

A chronic inflammatory state to a large extent explains sickle cell disease (SCD) pathophysiology. Nonetheless, the principal dysregulated factors affecting this major pathway and their mechanisms of action still have to be fully identified and elucidated. Integrating gene expression and genome-wide association study (GWAS) data analysis represents a novel approach to refining the identification of key mediators and functions in complex diseases. Here, we performed gene expression meta-analysis of five independent publicly available microarray datasets related to homozygous SS patients with SCD to identify a consensus SCD transcriptomic profile. The meta-analysis conducted using the MetaDE R package based on combining p values (maxP approach) identified 335 differentially expressed genes (DEGs; 224 upregulated and 111 downregulated). Functional gene set enrichment revealed the importance of several metabolic pathways, of innate immune responses, erythrocyte development, and hemostasis pathways. Advanced analyses of GWAS data generated within the framework of this study by means of the atSNP R package and SIFT tool identified 60 regulatory single-nucleotide polymorphisms (rSNPs) occurring in the promoter of 20 DEGs and a deleterious SNP, affecting CAMKK2 protein function. This novel database of candidate genes, transcription factors, and rSNPs associated with SCD provides new markers that may help to identify new therapeutic targets.

## Introduction

Sickle cell disease (SCD) is a rare hemoglobinopathy [1] characterized by high morbidity and mortality. It affects millions of people throughout the world, including 300 thousand newborns annually [2]. The disease represents a severe genetic blood disorder caused by a single mutation in β globin [3] that leads to a defective hemoglobin S (HBS) protein, which upon deoxygenation polymerizes rapidly [4] and gives red blood cells rigidity and sickle shape. Losing flexibility, the misshapen red blood cells obstruct blood flow, disrupt homeostasis, and promote chronic hemolysis. The released heme iron into plasma creates an oxidative microenvironment resulting in inflammation and endothelial-cell activation, leading to tissue damage in almost all organs and systems.

The SCD pathophysiology is largely explained by the chronic inflammatory state [5] and partly by the dysregulated pro- and antiapoptotic agents [6]. Nonetheless, the principal dysregulated factors in these major pathways and their mechanisms of action still need to be fully identified and elucidated.

Several technologies [7,8] have been developed to identify candidate markers and have highlighted potential molecular pathways related to SCD. Among the high-throughput genomic technologies that have contributed to the identification of candidate genes and to our understanding of complex interactions in multisystem diseases is the microarray technology. Indeed, a microarray is a gene expression profiling technology that is widely used to simultaneously assess the expression levels of thousands of genes [9] in different disease contexts. Such high-throughput technique is considered an important tool for clinical practice and for the development of diagnostics [10,11]. Thus, its massive use in biomedical studies in the last decade has resulted in a huge quantity of data for a large number of physiological states and disease conditions [12]. In parallel, the constant evolution of bioinformatics tools provided the scientific community with a more effective and reproducible meta-analysis workflow [13–20] allowing for better handling of differences in study design and platforms and providing enhancement of the analytical performance, which results in a more robust and reliable analysis of gene expression signatures [21–24].

On the other hand, a genome wide association study (GWAS) is an approach that involves rapid scanning of markers (genetic variants) across the genomes of different individuals [25]. It focuses on association between genetic variations such as single-nucleotide polymorphisms (SNPs) and a specific disease. Recent studies have shown that integrative approach combining GWAS and gene expression data is more effective than analyzing each data type individually [26–29]. Indeed, SNPs present in the promoter regions of differentially expressed genes (DEGs) and more specifically in regulatory elements [30] are likely to be regulatory SNPs (rSNPs) that could mediate the binding of critical transcription factors (TFs) and consequently alter the expression of target genes [31]. Therefore, joint identification of rSNPs and a gene expression meta-signature will potentially advance the understanding of the disease mechanisms [30,31].

In the present study, we performed a gene expression meta-analysis to identify a consensus transcriptomic profile related to SCD, and by means of GWAS data, we investigated the involvement of rSNPs in the expression of the identified key candidate genes.

## Materials and methods

### Search criteria and data collection

**Fig 1:**
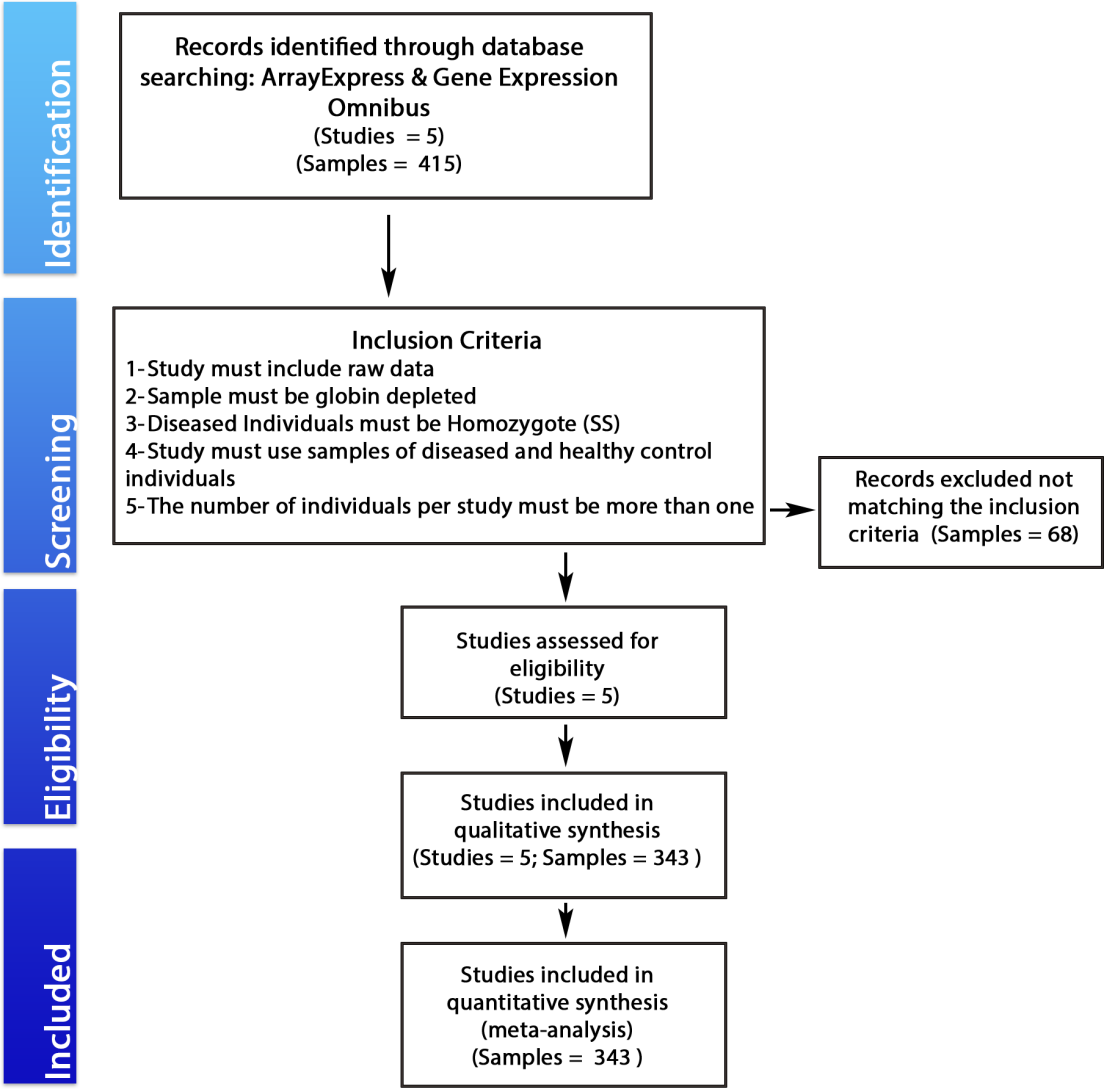
A PRISMA flow diagram of a systematic database search. The selection process of eligible microarray datasets for meta-analysis of the shared transcriptomic signatures between Sickle cell disease patients, according to Prisma 2009 flow diagram.

Analysis of the publicly available microarray datasets was carried out as per PRISMA guidelines [32] (S1 Table and **Fig 1**). A search involving a combination of the following keywords (“sickle cell disease” and “Homo sapiens”) was performed in two public microarray expression data repositories: NCBI Gene Expression Omnibus (GEO) Datasets [http://www.ncbi.nlm.nih.gov/geo/] and ArrayExpress database [http://www.ebi.ac.uk/arrayexpress/]. Studies that were publicly available by September 2016 were extracted. “Globin depleted” and “Homozygote SS” were set as sample inclusion criteria. Table 1 provides details on all the datasets included in this study.

**Table 1:** Characteristics of included individual microarray dataset.

GEO Accession Number	Tissue	Number of samples (Control/Cases)	Microarray Platform	Reference study
GSE11524	Peripheral blood	30(12/18)	Affymetrix Human Genome U133 plus 2.0	[33]
GSE16728	Peripheral blood	20(10/10)*	Affymetrix Human Genome U133 plus 2.1	[34]
GSE53441	Peripheral blood	34(10/24)	Affymetrix Human Genome U133 plus 2.2	[35]
GSE31757	Whole blood	8(3/5)	Affymetrix Human Exon 1.0 ST	[7]
GSE35007	Whole blood	251(61/190)	Illumina HumanHT-12 v4	[36]

Inclusion criteria: Only samples homozygotes SS and submitted to Globin reduction were selected.

*: Consists of two studies with two datasets in total

### Data preparation and quality control

Data preprocessing and meta-analysis were carried out using MetaQC [37] and MetaDE [18], respectively. These packages of the R suite were chosen for quality control (QC) and identification of differentially expressed genes (DEGs) in microarray meta-analysis. The input gene expression table was prepared from the downloaded raw data following MetaDE guidelines [18]. In brief, probe IDs of an individual study were annotated to retrieve their official gene symbols using Bioconductor. The interquartile range method was applied to select the probe ID with the largest interquartile range of expression values when multiple probes matched an identical gene symbol. Individual studies that contained samples from different populations were divided into substudies and treated as independent datasets during the meta-analysis. Afterwards, the processed data were analyzed with MetaQC to compute six QC measures: (1) internal homogeneity of coexpression structure among studies (IQC); (2) external consistency of coexpression structure correlating with a pathway database (EQC); (3) accuracy of DEG detection (AQCg) or pathway identification (AQCp); and (4) consistency of differential expression ranking of genes (CQCg) or pathways (CQCp). The datasets showing good performance on at least five QC criteria were retained. Table 2 presents the quantitative QC measures of each dataset.

**Table 2:** MetaQC quantitative quality control measures for gene expression data.

Dataset	IQC	EQC	CQCg	CQCp	AQCg	AQCp	Rank
GSE35007	2.53	3.7	301.34	307.65	145.57	220.18	2.17
GSE11524	5.33	3.6	148.75	307.65	101.43	210.4	2.5
GSE16728-A	3.58	3.7	145.79	307.65	78.46	157.71	3
GSE31757	3.97	1.17*	102.06	307.65	44.78	138.97	3.92
GSE53441	7.02	3.82	58.5	73.32	27.43	37.71	4
GSE16728-B	0.41*	3.6	33.06	212.76	18.27	88.93	5.42

Inclusion Criteria: Dataset with good performance in at least five quality control criteria

*: Low performance

### Differential expression analysis

DEG analysis was performed with Limma software package [38] for each dataset independently using an adjusted p value ≤0.05, based on Benjamini–Hochberg false discovery rate (FDR) and the moderated *t* test. In this meta-analysis, DEGs across diseases and healthy controls were selected by means of a maximum p value (maxP) [39]. This method combines p values and targets DEGs that have small p values in all studies (FDR < 0.05). Significantly up- and downregulated DEGs were defined as those that showed a fold change (FC) greater than 1.4 in either direction.

### Functional annotation and gene-regulatory network inference

The candidate genes identified via our meta-signature were used as input for different bioinformatics enrichment tools. Gene Ontology (GO) terms were identified in the TRANSPATH database [40] on the geneXplain platform [41]; a different pathway gene set libraries (KEGG Pathway, Reactome, and Wiki pathway), were requested by means of the EnrichR web tool [42]. The selection criterion for significantly enriched pathways and GO terms was a p value of 0.05 or less. Furthermore, for better result interpretation, we constructed a biological network describing the most significant over-represented GO biological process terms obtained at the previous step. In brief, we conducted two-sided (enrichment/depletion) hypergeometric distribution tests, with a p value significance level of ≤0.05, followed by the Bonferroni adjustment using the ClueGO plugin [43] of the Cytoscape [44] software.

Protein–protein (PP) interactions including physical and functional association across our set of genes were identified in stringdb 10.0 [45]. To gain more insights into post-transcriptional regulation of expression of DEGs, we used the complete list of our DEGs, their log FC, and the p value as input for the RNEA R [46] package to reconstruct a subnetwork of gene regulation.

### GWAS data integration

GWAS was carried out on 1,213 individuals (HbSS and HbSb0) and gave a collection of 15,153,765 SNPs and indels related to SCD [47]. From those we selected 1,000 SNPs (with the best p-value ranking) affecting the genes that we identified as DE by our meta-analysis. To identify the location of each SNP within the related candidate gene, we used the list of the 1,000 SNPs as input for the VariantAnnotation Bioconductor package with 2,000 as value representing the number of base pairs upstream of the 5’- gene end and 200 representing those downstream of the 3’- gene end. SNPs occurring in coding regions were used as input for SIFT (Sorting Intolerant from Tolerant) web server—able to identify nonsynonymous SNPs and predict the effect of coding variants on protein function. The remaining SNPs (occurring in non-coding regions) were used as input for atSNP (A:ffinity T:esting for regulatory SNP: s) R package [42] to predict the regulatory variants (rSNPs). This software later computes an affinity score for the genomic sequences around each SNP (±30 bp) against a library of TF motifs for both alleles (reference and SNP allele) using their corresponding position-weighted matrices (PWMs). SNPs with a significant change in the affinity scores between the reference and SNP alleles were then hypothesized as rSNPs. We selected ENCODE PWMs as a TF motif library and set a p value corrected by the Benjamini–Hochberg criterion in the rank test between alleles (pval_rank_bh) to less than 0.1 to select significant rSNPs.

**Fig 2:**
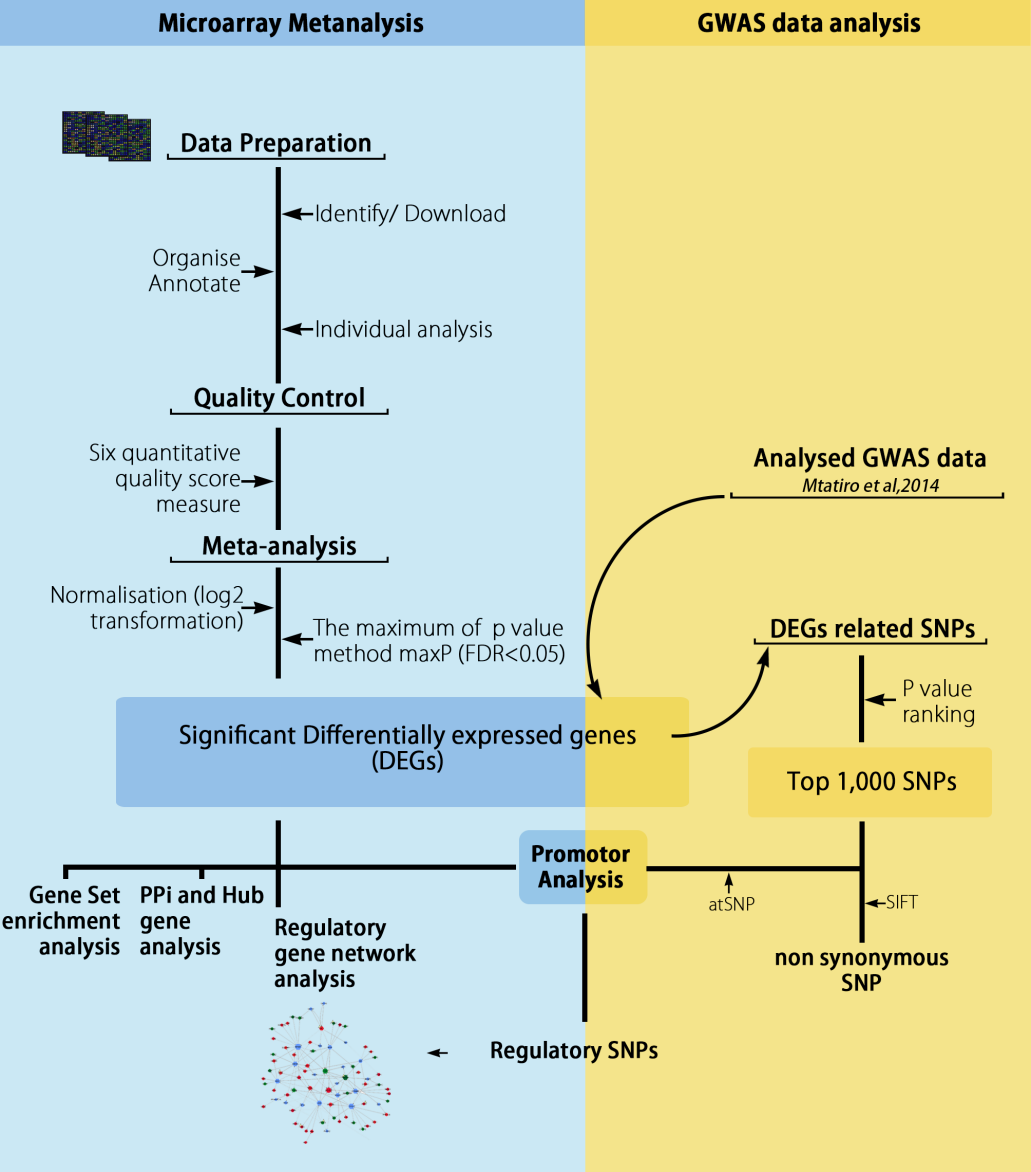
Workflow of microarray meta-analysis integrating GWAS data. A flow diagram depicting the process involved in the meta-analysis of the selected microarray datasets with integration of GWAS data.

The main steps of the bioinformatics workflow integrating microarray meta-analysis and GWAS data are described in **Fig 2**.

## Results

### Data mining and quality assessment

Five case-control studies (GSE11524, GSE16728, GSE53441, GSE31757 and GSE35007) satisfying the fixed inclusion criteria were selected for this meta-analysis. GSE16728 includes two cohorts; each cohort was regarded as a different dataset and was analyzed separately. Sample sources were either peripheral blood or whole blood subjected to globin mRNA depletion [48]. The retained studies included 343 samples representing a total of 247 homozygous SCD patients and 96 healthy controls. Table 1 provides detailed information about the datasets, sample types, study references, and the microarray platforms used here. All expression-processed raw data that yielded a high score at the QC step are presented in Table 2.

### Identification of the gene expression meta-signature

A shared transcriptional signature among SCD patients was identified by a meta-analysis of the retained datasets by combining p values according to the stringent maxP method [39]. The raw data were loaded into the R environment and analyzed using the R package MetaDE. Non-DEGs as well as those showing small variation and expression intensities across the majority of datasets and contributing to the FDR were filtered out. The statistical framework (maxP) identified highly significant biomarkers that are differentially expressed in all studies: 335 DEGs including 224 overexpressed and 111 underexpressed genes satisfying the significance threshold of FDR < 0.05 (see S2 Table).

The top 10 upregulated and top 10 downregulated DEGs are presented in Table 3, along with the average FC in expression. Among these, DnaJ heat shock protein family (Hsp40) member C6 (*DNAJC6*), ring finger protein 182 (*RNF182*), and carbonic anhydrase I (*CA1*) were the most significantly overexpressed genes, whereas Wntless Wnt ligand secretion mediator (*WLS*), Transcription factor 7-like 2 (T-cell specific, HMG-box) (*TCF7L2*), and G protein-coupled receptor 171 (*GPR171*) were the most underexpressed genes (Table 3).

**Table 3:** Top 20 shared DEGs identified in the meta-analysis ranked by average Log2FC.

Entrez Gene ID	HGNC Gene symbol	Gene Description	Average Log2FC	FDR
**Top 10 upregulated Genes**				
9829	*DNAJC6*	DnaJ heat shock protein family (Hsp40) member C6	4,938	0,017
221687	*RNF182*	Ring finger protein 182	4,003	0,002
759	*CA1*	carbonic anhydrase I	3,366	1,38E-18
3045	*HBD*	Hemoglobin subunit delta	3,294	1,38E-18
2994	*GYPB*	Glycophorin B (MNS blood group)	3,246	1,38E-18
9911	*TMCC2*	Transmembrane and coiled-coil domain family 2	3,166	1,38E-18
2993	*GYPA*	Glycophorin A (MNS blood group)	3,070	1,38E-18
8140	*SLC7A5*	Solute carrier family 7 (amino acid transporter light chain, L system), member 5	2,999	0,0004
66008	*TRAK2*	Trafficking protein, kinesin binding 2	2,985	0,023
493856	*CISD2*	CDGSH iron sulfur domain 2	2,980	1,38E-18
**Top 10 downregulated Genes**				
79971	*WLS*	Wntless Wnt ligand secretion mediator	-1,766	0,036
6934	*TCF7L2*	Transcription factor 7-like 2 (T-cell specific, HMG-box)	-1,562	0,016
29909	*GPR171*	G protein-coupled receptor 171	-1,523	0,027
79668	*PARP8*	Poly(ADP-ribose) polymerase family member 8	-1,344	0,002
64167	*ERAP2*	Endoplasmic reticulum aminopeptidase 2	-1,343	0,048
91526	*ANKRD44*	Ankyrin repeat domain 44	-1,300	0,001
5788	*PTPRC*	Protein tyrosine phosphatase, receptor type, C	-1,275	0,005
1362	*CPD*	Carboxypeptidase D	-1,209	0,030
50852	*TRAT1*	T cell receptor associated transmembrane adaptor 1	-1,169	0,009
23224	*SYNE2*	Spectrin repeat containing, nuclear envelope 2	-1,159	0,047

### Network construction

**Fig 3:**
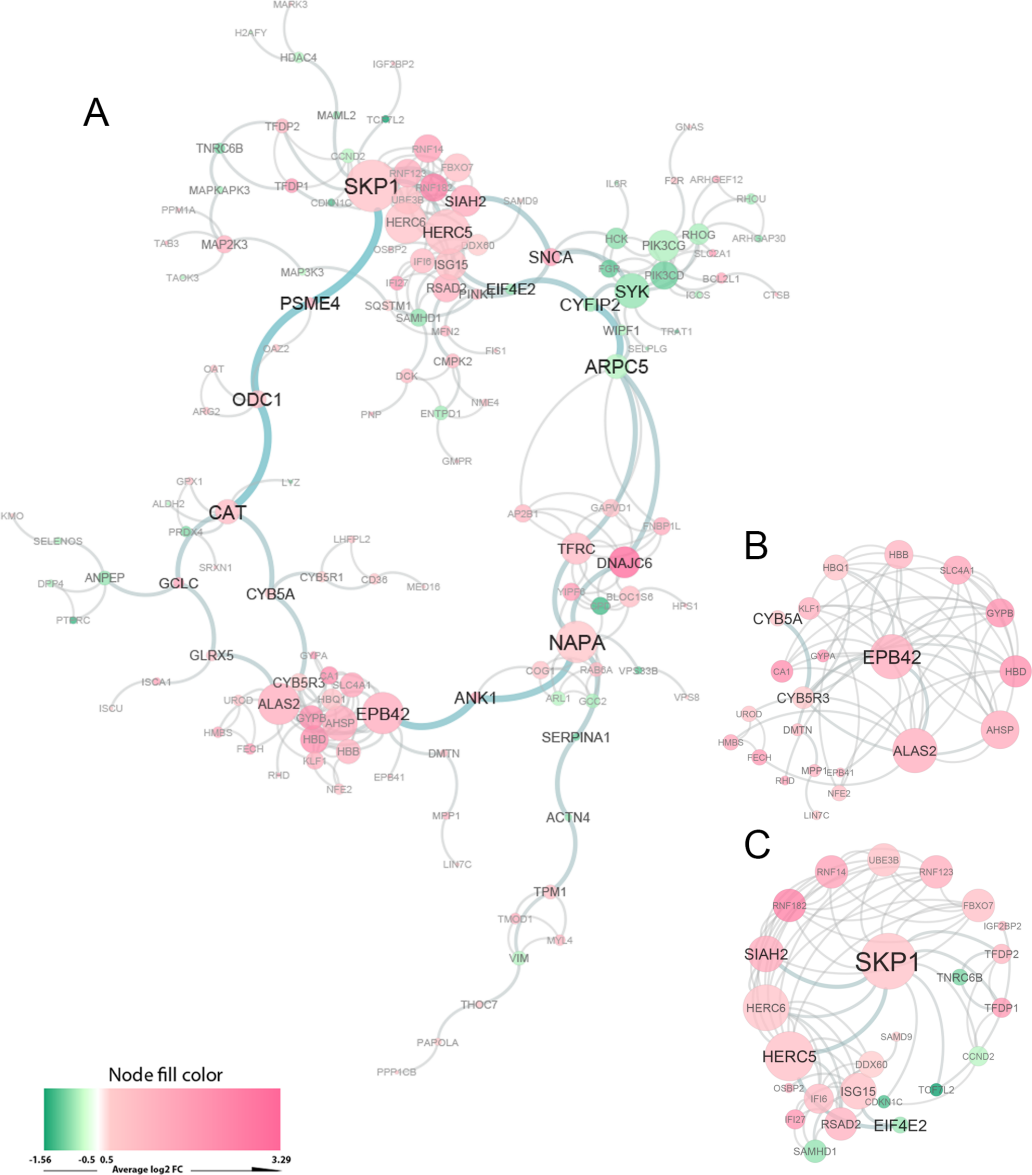
Network-based meta-analysis of hub genes. **A:** Protein interaction network analysis indicates a central role for SKP1, NAPA, EPB42, and ARPC5 in SCD anemia. All 335 genes served as input for the STRING database with the high confidence interaction score 0.7, and a network was built by means of Cytoscape. The network topology was analyzed by the Cytoscape NetworkAnalyzer tool, and then network topology measures such as the degree (represented by the node size scale), betweenness (represented by police size scale), closeness centrality, and clustering coefficient were calculated. **B and C:** The top-ranked subnetwork identified by the OH-PIN algorithm (threshold: 2, overlapping score 0.5) using CytoCluster (a Cytoscape plugin).

PP interaction analysis was conducted to identify the key hub genes among our 335 DEGs. Using the STRING database [45], we generated a PP interaction network among 175 nodes with a high confidence score of 0.7. For better visualization of the network, we extracted the most connected components that represent the 139 major nodes of the network. These included 139 proteins and 278 edges representing the interactions within **Fig 3A.** Based on network topology measures, a list of top 10 hub genes was compiled (see S3 Table). Four key hub genes that showed a higher score in both degree and betweenness-centrality metrics are coding for S-phase kinase-associated protein 1 (SKP1; degree = 15, betweenness centrality = 0.35), HECT and RLD domain-containing E3 ubiquitin protein ligase 5 (HERC5; degree = 13, betweenness centrality = 0.14), NSF attachment protein alpha (NAPA; degree = 12, betweenness centrality = 0.32), and erythrocyte membrane protein band 4.2 (EPB42; degree = 12, betweenness centrality = 0.22). Uploading the 139 proteins via the CytoCluster Cytoscape plugin extracted the top-ranked clusters as a subnetwork. These included SKP1 (25 nodes and 73 edges) and EPB42 (22 nodes and 52 edges). Both subnetworks are independent protein complexes with their interacting nodes likely to work collectively to perform biological functions listed in Fig 3B and 3C.

**Fig 4:**
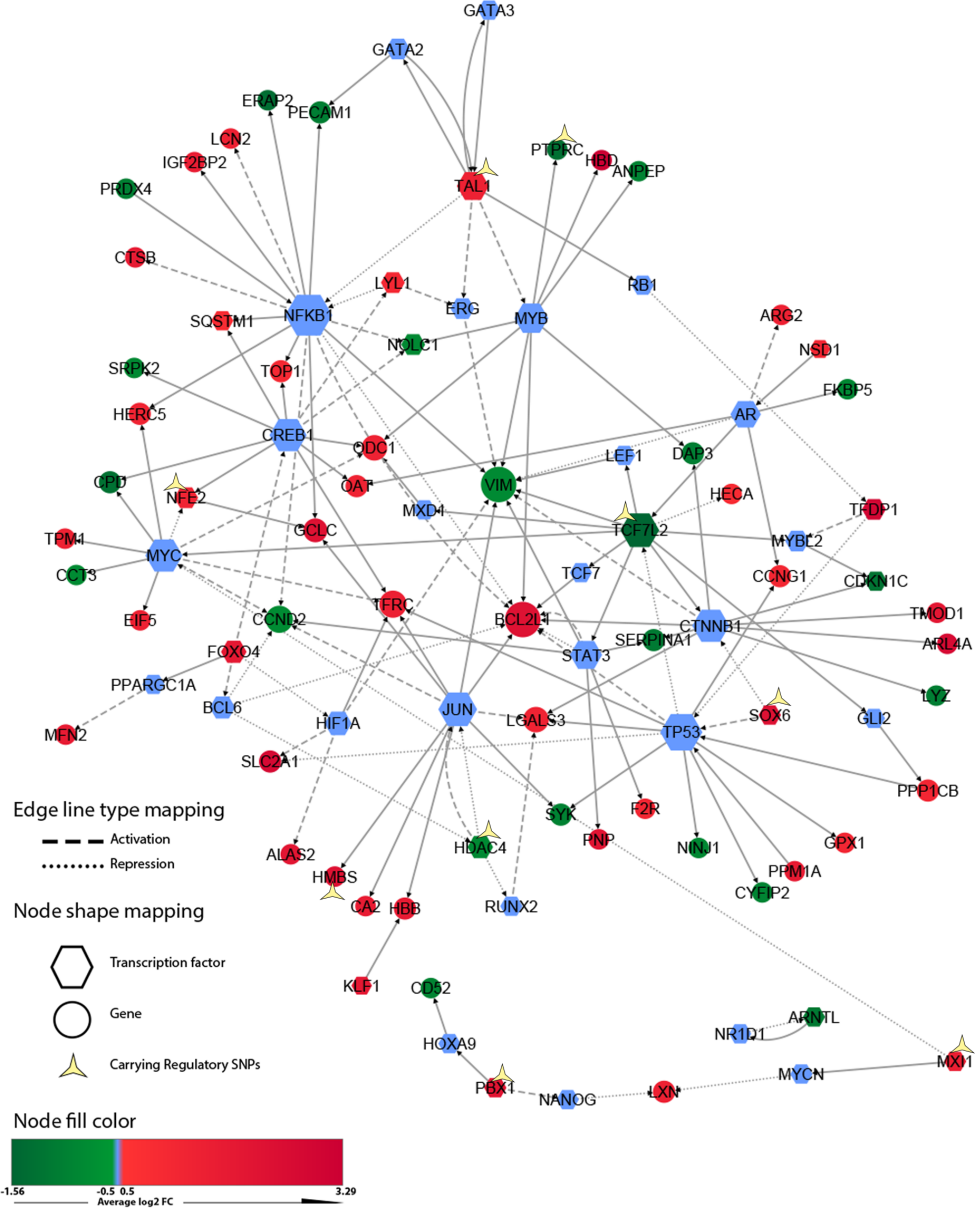
Transcriptional regulatory subnetwork based on microarray meta-analysis. Regulatory network analysis was performed using RNEA R/package to determine the regulation complexes upstream of DEGs identified in the meta-analysis. Genes carrying in their promoter, a significant regulatory SNPs are marked by a yellow star. Each node represents a DEG or enriched transcription factor, depending on their shapes. The node size indicate greater significance of the enrichment. The edges reflect the relationships between the nodes.

To gain insight into the regulatory system upstream of the identified DEGs, we employed the Regulatory Network Enrichment Analysis (RNEA) bioinformatics tool that extracts lists of prioritized TFs. A regulatory subnetwork showing the interaction of activated regulators with their target genes and with one another is shown in **Fig 4**.

### Identification of regulatory and nonsynonymous SNPs

Identifying potential rSNPs is crucial for understanding disease mechanisms. From a subset of the most significant 1,000 DEGs associated SNPs (retrieved from GWAS data analysis based on P-value) and using the atSNP R package [15], we evaluated the regulatory potential of 789 SNPs (occurring in non-coding regions) by comparing them with a motif library of 2,065 PWMs from the ENCODE project [16]. The analysis revealed a total of 60 significant rSNPs that affect gene regulation of 20 candidate disease genes out of 335 DEGs. These SNPs affect binding affinity of TFs for their target promoter (full results are in S4 Table). Besides, we identified seven TFs (Fig 4) carrying rSNPs such as NFE2, which plays a prominent role in the hematopoietic stem cell differentiation pathway.

We next evaluated the functional impact of the remaining coding variants (211 SNPs out of 1,000 SNPs) in the affected proteins. According to SIFT, one nonsynonymous SNP (rs1132780) was predicted as damaging with a significant score of 0.042 (results are in S5 Table). Rs1132780 occurs in the coding region of the calcium/calmodulin-dependent protein kinase kinase 2 (CAMKK2) gene and affects all its catalytic domain isoforms with an amino acid change from arginine to cysteine at position 363 of the protein.

### Gene set enrichment analysis for identification of over-represented biological pathways and GO terms

**Fig 5:**
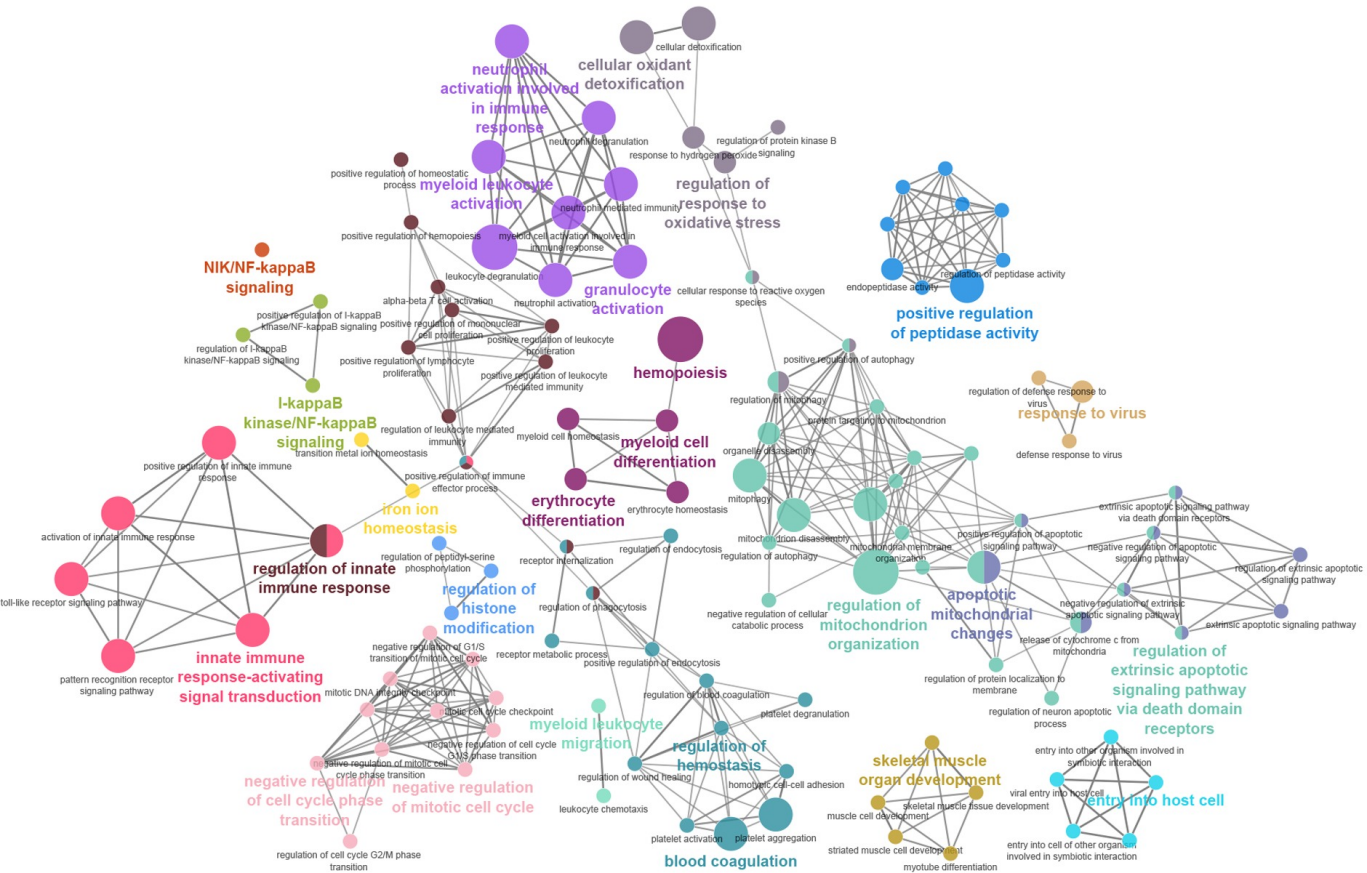
Over-representation of pathways and GO categories in biological networks identified by the meta-analysis. Network representation of an enriched pathway integrating biological processes on the DEG list according to the ClueGO Cytoscape plugin. Hypergeometric (right-handed) enrichment distribution tests were conducted with a p value significance level of ≤0.05, followed by the Bonferroni adjustment for the terms, and thus leading term groups were selected based on the highest significance. Each node represents a biological process. The edges reflect the relationships between the terms based on the similarity of their associated genes. The node size and deeper color indicate greater significance of the enrichment.

Enriched GO biological process terms and biological pathways associated with our complete list of DEGs and showing a p value <0.05 were identified by means of both geneXplain and EnrichR tools (full results are in S6 Table). Among the top significant terms identified were those associated with an innate immune response, oxidative stress, hemostasis, and hemopoiesis (**Fig 5**). Relevant GO terms identified only on the basis of the list of DEGs carrying rSNPs were associated mainly with B-cell proliferation, T-cell differentiation, B-cell activation involved in an immune response, and T-cell lineage commitment.

## Discussion

SCD is a monogenic disorder characterized by chronic hemolytic anemia and episodic vaso-occlusion. These major pathological signs are primarily triggered by the polymerization of defective hemoglobin S [49] and end with a sustained inflammatory response and tissue damage. Multiple cell types are involved in the chronic inflammatory state and are associated with SCD severity, including leukocytes, activated monocytes, neutrophils, and platelets [50–52]. In addition, SCD patients show higher levels of several molecules that promote inflammation, including heme and proinflammatory cytokines, such as TNF-α, IL-1β, and IL-8 [53–55], which enhance the vaso-occlusive process [53,56] and aggravate the disease.

The inflammatory response thus plays a prominent role in SCD pathophysiology [51] and needs to be resolved to help control the disease and prevent the subsequent damage to organs. Nevertheless, the mechanisms that determine the appearance of the inflammatory response and its persistence (which exacerbates the symptoms of SCD patients) and the factors involved still need to be clarified.

Here, we set up a full complete 'omics bioinformatics pipeline to further delineate the relation between SCD patients’ transcriptomics profile and the pathophysiology of the disease focusing especially on the inflammatory response.

Several individual microarray-based gene expression studies related to SCD have been published in recent years and were made available for public reuse. On the other hand, the small number of individuals analyzed in each study is an obstacle for the identification of a consensus set of DEGs. A meta-analysis of the available data should reduce study bias, increase statistical power, and improve the overall biological-process understanding [57,58], which may lead to therapeutic-target discovery [59,60].

In the present study, we not only increased the sample size of individuals subjected to the meta-analysis but also overcame the heterogeneity of the disease expression across both SCD patients and individual datasets by means of a bioinformatics approach that takes into account the between-study and within-study variation.

Our meta-analysis, using all public eligible SCD raw data [7,33–36] produced a consensus shared DEG profile. Indeed, we identified 335 DEGs, which include 224 upregulated and 111 downregulated genes having FDR < 0.05. Among these, we found the already known set of genes previously reported to be associated with SCD, e.g., *KLF1* (Krüppel-like factor 1) [61,62] and *HBD* (hemoglobin subunit delta), as well as more than 130 candidate genes found to be differentially expressed by a preceding meta-analysis despite a different statistical framework that was applied to only two datasets [63].

The gene set enrichment and pathway analysis of our DEG set revealed the importance of **innate immunity** (regulation and activation of innate immune response “GO:0002218, GO:0045088,” innate immune system “R-HSA-168249,” interferon alpha/beta signaling “R-HSA-909733,” the type I interferon signaling pathway “GO:0060337,” and the IL-6 signaling pathway, *Homo sapiens* “WP364”), **hemostasis** (blood coagulation “GO:0007596,” platelet activation “hsa04611,” and positive regulation of the MAPK cascade “GO:0043410”), a **response to stress** (oxidative stress “WP408,” glutathione metabolism “WP100”) and **hemopoiesis** (erythrocyte development “GO:0048821,” myeloid cell development “GO:0061515”) pathways. **Heme biosynthesis** and **apoptosis** were also found among the significant biological terms (a complete list is provided in S6 Table).

The results of the PP interaction analysis supported those obtained by the gene enrichment analysis. Indeed, among the top predicted hub genes sorted by degree and betweenness centrality, we found genes that play a prominent role in innate immunity pathways (*HERC5*, *HERC6*), hemopoiesis (*AHSP*), and heme biosynthesis (*ALAS2*). The latter (5'-aminolevulinate synthase) encodes a major regulatory enzyme in erythrocytes [64]. This protein is the rate-limiting enzyme in heme synthesis and contributes to regulation of heme (iron-containing molecule) synthesis preventing free-iron accumulation and subsequent organ damage [65].

Our findings showing the increase in *ALAS2* transcription (logFC = 1.9) are in agreement with the previously reported higher heme concentration measured in SCD patients’ groups (steady state and crisis) when compared with healthy individuals [66]. This high heme level has been shown to induce inflammation and to increase vascular permeability, adhesion molecule expression, and leukocyte recruitment [67,68] and has been suggested to contribute to severe clinical manifestations of SCD [69]. Accordingly, we did not notice either the transcription of heme oxygenase gene (*HMOX1*; coding for the enzyme regulating catalytic cleavage of heme groups) or the expression of the master TF *NRF2* (its protein product regulates the transcription of most of antioxidant genes). This observation is in line with another study showing that a large proportion of SCD patients have a relatively modest HMOX1 plasma concentration due in part to a hyporesponsive *HMOX1* promoter [70]. Nevertheless, our results revealed transcriptional induction of other antioxidant genes such as *GPX1*, *GCLC*, *CAT*, *PINK1*, *SESN3*, *UBQLN1*, *CD36*, and *SNCA*.

Carbon monoxide (CO), one of the products of HMOX1, has been widely studied for its therapeutic potential [71–75]. Indeed, administration of CO and biliverdin inhibits vascular inflammation and vaso-occlusion in mouse models of SCD [72]. Furthermore, HMOX1 induction has had a beneficial effect in several pathological conditions [76] including chronic nephropathy [77]: an SCD-related pathology. HMOX1 inducers may thus help to decrease heme concentration and consequently reduce the inflammatory responses that worsen the patients’ symptoms; i.e., HMOX1 inducers may be beneficial to SCD patients.

For a better understanding of the transcriptional mechanisms that regulate our DEGs, we constructed a gene-regulatory subnetwork associated with SCD (**Fig 4**). According to the network topology analysis, the nuclear factor-kappa B (NF-κB) remained the top hub-enriched TF. NF-κB regulates several physiological responses, including transcription of a large set of proinflammatory-cytokine genes [78] usually upregulated in SCD [53–55]. Our results indicate the increased transcription of various proinflammatory-cytokine genes including *IL-6*, *IL-12*, and *IL-18* and cytokine receptor genes such as *IL-17RD* and *IL-17RC* even if the increased expression of these transcripts was below the cutoff.

At the same time, using GWAS data, from the identified polymorphisms we extracted the rSNPs occurring in the promoter region of our DEGs and gained an important insight into the regulatory complexes governing these gene expression patterns. Our analysis revealed the occurrence of 60 rSNPs in promoter DNA sequences of 20 genes (S4 Table), including *NFE2*, *HDAC4*, *PI3K*δ, and *PI3K*γ.

Among the top significant enriched GO terms and biological pathways associated with the subset of these 20 candidate disease-associated genes were those primarily related to T-cell activation/differentiation; alpha-beta, gamma-delta, and regulatory T-cell differentiation; T-cell lineage commitment; and B-cell proliferation or activation. This result is highly consistent with the alteration of a lymphocyte count [79], phenotype, and function associated with SCD [80]. This alteration in the acquired immunity may explain the increased risk of severe bacterial infections among these patients [81,82]. Indeed, different studies have shown that SCD patients have a decreased T-lymphocyte count and increased B-lymphocyte count either at baseline or in acute crisis [83]. Of note, T-cell lymphopenia–associated inflammatory responses have been previously linked to the inactivation of *PI3K*δ and *PI3K*γ [84], genes of two PI3K isoforms that we found to be significantly downregulated and to carry rSNPs (log_2_FC: -0.94 and -0.56, respectively) in this analysis. This finding suggests that the downregulation of these kinase genes, predominantly expressed in the hematopoietic system [85], may be implicated in the profound lymphopenia observed in SCD patients.

**Fig 6:**
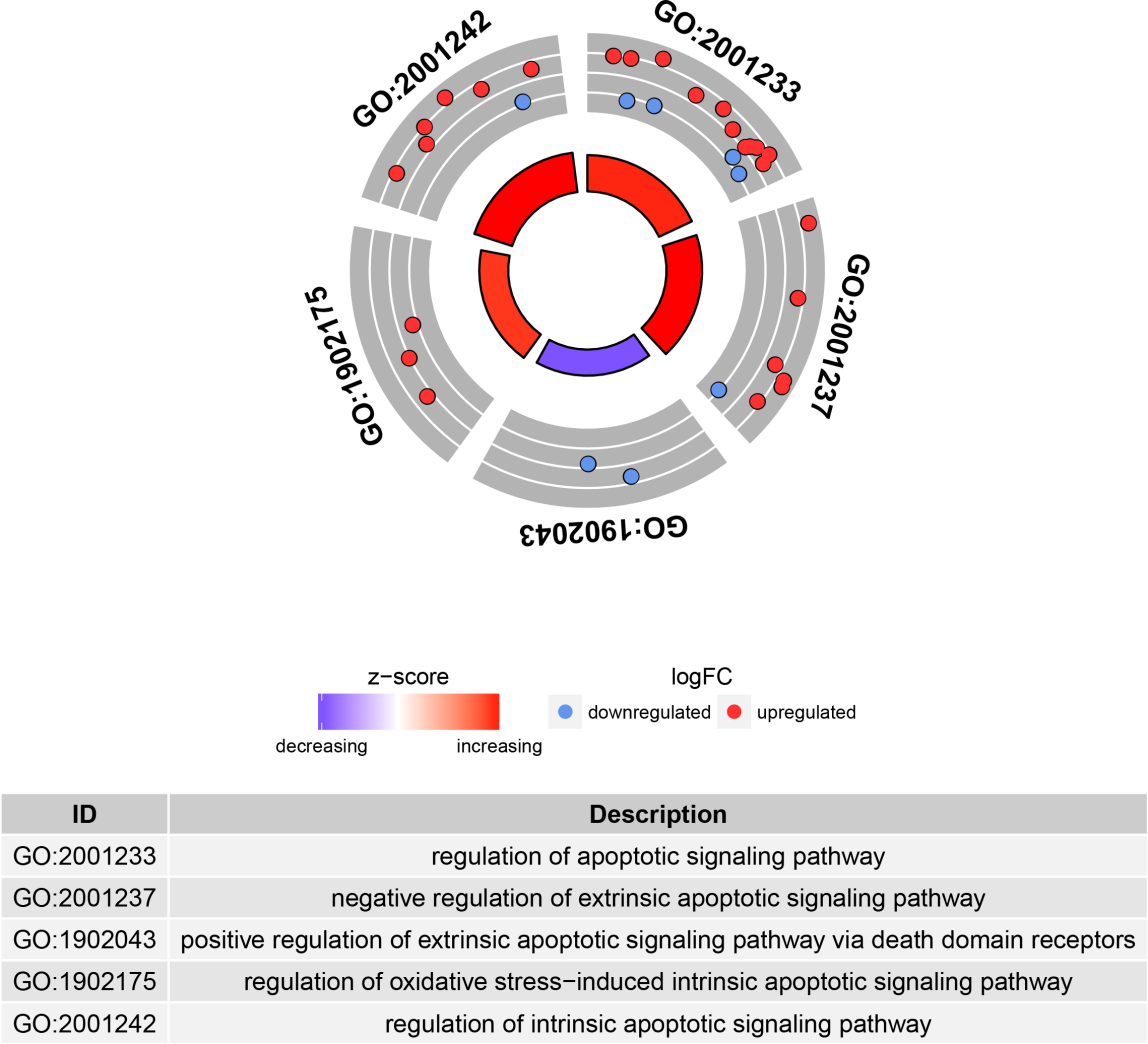
Downregulated and upregulated DEGs involved in apoptosis pathways. The inner ring is a bar plot where the height of the bar indicates the significance of the term (−log_10_ adjusted *p* value), and color corresponds to the *z*-score. The outer ring displays scatterplots of the expression levels (log2FC) for the genes in each term.

Our gene-regulatory network also showed significant overexpression of the TF nuclear factor-erythroid 2 (*NFE2*). The expression of this TF is activated by CREB1 and repressed by MYC TFs. Promoter analysis revealed that *NFE2* is affected by five rSNPs including rs35702801 and rs34155291 (S7 Table) that are predicted to disrupt several MYC motifs but not to affect the affinity of CREB1 binding. These rSNPs by altering TF MYC binding may hence explain the overexpression of *NFE2* observed in SCD patients. Some studies have shown that elevated expression of *NFE2* modulates proinflammatory cytokine IL-8 expression in myeloid cells [86] and causes a high neutrophil count in a murine model [87]. We suggest here that overexpression of *NFE2* may contribute to the neutrophilia associated with the severity of SCD. This neutrophilia may be—as suggested by others—accentuated by the alteration of neutrophil apoptosis, which leads to neutrophil accumulation in blood [88]. Accordingly, our results reveal downregulation of the DEGs involved in the positive (“GO:1902043”) and negative (“GO:2001237”) regulation of the extrinsic apoptotic signaling pathway (**Fig 6**).

Additionally, the GWAS revealed a nonsynonymous SNP occurring in *CAMKK2*’s protein-coding region (S5 Table), transforming arginine to cysteine at position 363 present in all CAMKK2 protein isoforms including the active isoforms 1, 2, and 3. Thus, besides the downregulation, the amino acid change affecting the catalytic domain was predicted by the SIFT analysis to be a deleterious SNP with a significant damage score (0.041). CAMKK2 belongs to a family of Ca^2+^/CaM-dependent, highly versatile serine/threonine kinases that catalyze the phosphorylation of (and have high affinity for) mainly three substrates: CAMK1, CAMK4, and AKT. CAMKK2 was reported to be exclusively expressed in the myeloid lineage [89] and participates in the regulation of several relevant physiological and pathophysiological processes, including hematopoiesis, cancer, inflammation, and immune responses. It was recently reported that under stressful conditions in immune cells, the loss of CAMKK2 promotes differentiation (both in bone marrow and peripheral-blood cells) of common myeloid progenitors toward granulocyte and monocyte precursor cells rather than megakaryocytes or erythrocytes [89]. This nonsynonymous SNP occurring in *CAMKK2*’s protein-coding region may thus also contribute to the increase in the neutrophil number (the most abundant type of granulocytes). Yet, the granulopoiesis state can be reversed by the re-expression of *CAMKK2*. Further research is needed to determine whether the CAMKK2 protein present in the blood of SCD patients shows an altered activity.

In conclusion, besides previously reported genes, our meta-signature contains a new subset of genes identified for the first time as associated with SCD. Among these, *ALAS2* belonging to heme metabolism is well known to be associated with SCD, as are PI3K delta and gamma isoforms involved in T-cell differentiation. The present analysis yielded a large database of rSNPs and candidate genes that may be helpful for future studies dealing with the pathogenesis and complexity of SCD.

## Supporting information

S1 Table Preferred reporting items for systematic reviews and meta-analyses (PRISMA). Guidelines Checklist.
(PDF)

S2 Table Complete list of differentially-expressed genes obtained from the meta-analysis. (XLSX)

S3 Table Top 10 hub genes. (XLSX)

S4 Table The rSNPs located in the promoter region and altering TF-binding sites’ affinity identified by the atSNP R package. (XLSX)

S5 Table Evaluation of the functional impact of the coding variants in the affected candidate gene proteins using Sift software. (XLSX)

S6 Table Gene set enrichment analyses of biological processes and metabolic pathways using multiple tools. (XLSX)

S7 Table rSNPs located in the promoter region of NFE2 and altering MYC-binding sites’ affinity. (XLSX)
